# Comprehensive Assessment of the Vecta46 Intermediate Catheter for Neurovascular Interventions

**DOI:** 10.7759/cureus.90577

**Published:** 2025-08-20

**Authors:** Alan Napole, Georgios Sioutas, Pierce Davis, Chethan Reddy, Saarang Patel, Sandeep Kandregula, Sneha S Mannam, Rashad Jabarkheel, Kyle W Scott, Oleg Shekhtman, Jan-Karl Burkhardt, Visish M Srinivasan

**Affiliations:** 1 Neurosurgery, University of Pennsylvania Perelman School of Medicine, Philadelphia, USA

**Keywords:** endovascular, neuroendovascular surgery, neurosurgery, neurovascular, vecta46

## Abstract

Background

Advancements in neuroendovascular surgery with sophisticated catheters have enhanced outcomes in complex procedures. The FDA-approved Vecta46 intermediate catheter (IMC) by Stryker Corporation (Kalamazoo, MI) is used in various neurovascular interventions. Since its approval, the Vecta46 IMC has not been comprehensively studied. This study evaluates its safety, versatility, and efficacy in real-world multi-pathology applications.

Methods

We retrospectively reviewed 53 patients who underwent endovascular procedures with the Vecta46 IMC. We collected and analyzed demographic details, baseline characteristics, procedural specifics, and clinical outcomes.

Results

Among 53 cases, common comorbidities included hypertension (n = 25, 47.2%) and hyperlipidemia (n = 22, 41.5%). Antiplatelet use was noted in 43.4% (n = 23), and anticoagulant use in 13.2% (n = 7). Aneurysm treatments accounted for 56.6% (n = 30) of cases, followed by middle meningeal artery (MMA) embolization for chronic subdural hematomas (cSDHs) (18.9%) (n = 10), stroke thrombectomies (11.3%) (n = 6), Onyx embolization for dural arteriovenous fistulas (dAVFs) (7.5%) (n = 4), and arteriovenous malformations (AVMs) (5.7%) (n = 3). Flow diversion was the most common aneurysm treatment (56.7%) (n = 17). Most procedures were transradial (79.2%) (n = 42), primarily on the right side (88.7%) (n = 47), with verapamil used in 75.5% (n = 40). The mean number of vessels selected was 3.2±1.9, procedure duration was 102.4±66.8 minutes, fluoroscopy time was 43.6±31.1 minutes, contrast dose was 80.5±31.6 mL, and reference air kerma was 2114.0±1349.4 mGy. The Vecta46 was successfully navigated to the target vessel in 96.2% (n = 51) of cases. Intraprocedural complications included a nontarget vessel embolization, a thromboembolic event, and a non-flow-limiting focal dissection. No hemorrhagic complications or vessel irregularities occurred in the Vecta46 region. Post-procedural complications were minimal, with only forearm edema and an access site hematoma.

Conclusions

The Vecta46 catheter is an effective and safe tool for endovascular procedures, demonstrating versatility and reliability in neurovascular interventions.

## Introduction

The field of neuroendovascular surgery has significantly advanced in the past decade, particularly with the development of sophisticated catheter systems. Techniques such as intrasaccular embolization and flow diversion have revolutionized intracranial aneurysm treatment, facilitating the management of complex and wide-necked aneurysms with better outcomes [1]. This shift in treatment paradigm has been well-documented in the neuroendovascular literature, emphasizing the significant impact of these advancements on patient outcomes [2]. With the development and 510(k) FDA approval of 301 neuroendovascular devices between 2000 and 2022, it is crucial that further studies characterize their efficacy and versatility in neuroendovascular surgery [3].

The FDA-approved Vecta46 intermediate catheter (IMC), developed by Stryker Corporation (Kalamazoo, MI), exemplifies these advancements with features such as a distal flex zone, atraumatic tip, and hydrophilic coating, as well as facilitating and guiding interventional devices into selected blood vessels. These features enhance its navigability and compatibility with various microcatheters and guidewires, making it suitable for a wide range of neurovascular interventions. These interventions include intracranial aneurysm treatments, middle meningeal artery (MMA) embolization for chronic subdural hematomas (cSDHs), stroke thrombectomies, embolization for dural arteriovenous fistulas (dAVFs), and treatment of arteriovenous malformations (AVMs). These design enhancements are crucial for effectively reaching distal occlusions and providing stable support during intricate procedures. Additionally, the Vecta46's large lumen and aspiration capabilities also make it a valuable tool for mechanical thrombectomy, a similar characteristic shared with its predicate device, the Vecta71 and Vecta74 catheters.

Since its 510(k) FDA approval in August of 2021, the Vecta46 IMC has not yet been comprehensively investigated. Here, we demonstrate the safety, versatility, and efficacy of the Vecta46 IMC across various neurovascular interventions.

## Materials and methods

Study design

We conducted a comprehensive retrospective review of 53 consecutive patients aged 18 and older who underwent endovascular procedures utilizing the Vecta46 IMC from August 2023 to May 2024 in our tertiary cerebrovascular center. Open surgery cases or emergent open surgery conversions were excluded from this study. In all cases, Vecta46 was the IMC of choice and not a backup chosen after failure of a different device. The study was approved by the Penn Medicine Institutional Review Board (approval number: 850267), and informed consent was waived due to the retrospective nature of the study. 

Data collection

For each patient, demographic details, baseline characteristics, procedural details, and clinical outcomes were collected. The data included age, sex, past medical history of hypertension, hyperlipidemia, coronary artery disease, diabetes mellitus, and peripheral vascular diseases, home anti-thrombotic therapies, pathology and laterality, endovascular modality of treatment, vessel selection and catheterization, and procedural details such as access site, access laterality, duration, contrast dose, fluoroscopy time, reference air kerma, verapamil use, and intra- and post-procedural complications.

Statistical analysis

Mean and standard deviation (SD) were generally reported for continuous data. Categorical variables were described using frequencies.

## Results

Patient demographics 

The patient characteristics of all included patients are outlined in Table 1. Vecta46 was utilized in 53 cases. Patients ranged in age from 22 to 86 years, with an average age of 63±12.8 years. Of these patients, 31 (58.5%) were female and 22 (41.5%) were male. The most common comorbidities were hypertension (47.2%) (n = 25) and hyperlipidemia (41.5%) (n = 22). Antiplatelet use was noted in 43.4% (n = 23) and anticoagulant use in 13.2% of cases (n = 7). 

**Table 1:** Patient characteristics (n = 53) Table displaying patient characteristics for 53 included cases.

Variables	N (%)
Age, years (mean ± SD)	63.0 ± 12.8
Sex	
Male	22 (41.5%)
Female	31 (58.5%)
Medical history	
Hypertension	25 (47.2%)
Hyperlipidemia	22 (41.5%)
Coronary artery disease	7 (13.2%)
Diabetes mellitus	6 (11.3%)
Peripheral vascular disease	3 (5.7%)
Antiplatelet and anticoagulant medications on admission	
Antiplatelets medication	23 (43.4%)
Anticoagulant medication	7 (13.2%)

Procedural characteristics

**Figure 1:**
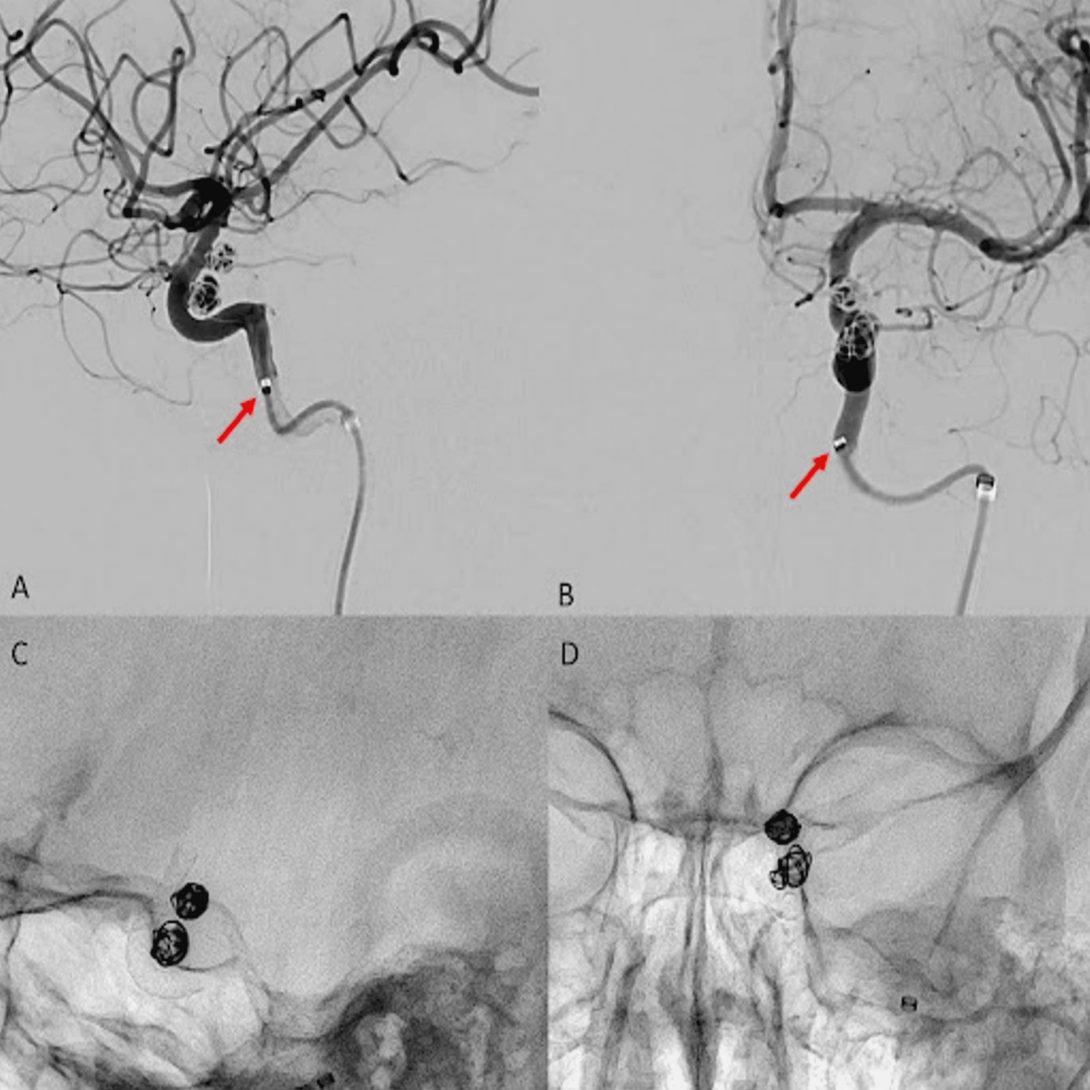
Successful coil embolization (A-D) Successful coil embolization of two adjacent left ophthalmic segment aneurysms along with the flow diverter (Surpass Evolve (Stryker Corporation)); (A, B) Vecta46 intermediate catheter pointed by the red arrows after the coiling phase before flow diverter placement.

The procedural characteristics of all included patients are outlined in Table 2. Aneurysm treatments comprised 56.6% of cases (n = 30), followed by MMA embolization for cSDH (18.9%) (n = 10), stroke thrombectomies (11.3%) (n = 6), Onyx embolization for dAVF (7.5%) (n = 4), and AVMs (5.7%) (n = 3). For aneurysm treatment, flow diversion was most common (56.7%) (n = 17), followed by simple coiling (13.3%) (n = 4), Woven EndoBridge (WEB; MicroVention, Inc., Aliso Viejo, CA) (13.3%) (n = 4), stent-assisted coiling (10.0%) (n = 3), contour (10.0%) (n = 3), and embolization (3.3%) (n = 1). Most procedures were transradial (79.2%) (n = 42), with 88.7% using the right radial artery (n = 47), and verapamil was used in 75.5% (n = 40). The mean number of vessels selected (excluding access site) was 3.2±1.9, with a mean procedure duration of 102.4±66.8 minutes, fluoroscopy time of 43.6±31.1 minutes, contrast dose of 80.5±31.6 mL, and reference air kerma of 2114.0±1349.4 mGy. Two transfemoral and one transradial conversions were necessary. The Vecta46 IMC was successfully navigated to the target vessel in 51 cases (96.2%), as shown in Figure 1. Unsuccessful attempts with the Vecta46 IMC were resolved by converting to either the AXS Catalyst 5 (Stryker Corporation) or Zoom 35 catheter (Imperative Care, Inc., Campbell, CA). Intraprocedural complications included embolization of nontargeted vessels (one case), a treated thromboembolic event (one case), and a non-flow-limiting focal dissection (one case). No hemorrhagic complications, vessel irregularities, vasospasms, or dissections occurred in the regions catheterized by Vecta46 IMC. Post-procedural complications were minimal, with one case of forearm edema and one case of a hematoma at the access site. 

**Table 2:** Procedural characteristics (n = 53 cases) cSDH: chronic subdural hematoma; dAVF: dural arteriovenous fistula; AVM: arteriovenous malformations; ICA: internal carotid artery; MMA: middle meningeal artery; MCA: middle cerebral artery; ACoA: anterior communicating artery; BA: basilar artery; SCA: superior cerebellar artery; VA: vertebral artery; OphA: ophthalmic artery; PCA: posterior cerebral artery; PCoA: posterior communicating artery; PICA: posterior inferior cerebellar artery; OA: occipital artery; STA: superficial temporal artery

Variables	N (%)
Laterality of pathology	
Right	20 (37.7%)
Left	29 (54.7%)
Middle	4 (7.5%)
Pathology	
Aneurysm	30 (56.6%)
cSDH	10 (18.9%)
Stroke	6 (11.3%)
dAVF	4 (7.5%)
AVM	3 (5.7%)
Treatment	
Aneurysm	
Flow diversion	17 (56.7%)
Simple coiling	4 (13.3%)
WEB	4 (13.3%)
Stent-assisted coiling	3 (10.0%)
Contour	3 (10.0%)
Balloon-assisted coiling	1 (3.3%)
Embolization	1 (3.3%)
cSDH	
Embolization	10 (100%)
Stroke	
Thrombectomy	6 (100%)
dAVF	
Embolization	4 (100%)
AVM	
Embolization	3 (100%)
Mean number of vessels selected (excluding radial or femoral)	3.2 ± 1.9
Vessels selected for Intervention	
ICA	11 (20.8%)
MMA	10 (18.9%)
MCA	9 (17.0%)
ACoA	6 (11.3%)
BA	3 (5.7%)
SCA	3 (5.7%)
VA	3 (5.7%)
OphA	3 (5.7%)
PCA	2 (3.8%)
PCoA	1 (1.9%)
PICA	1 (1.9%)
OA	1 (1.9%)
Maxillary artery	1 (1.9%)
STA	1 (1.9%)
Procedural details	
Access site	
Femoral	12.0 (22.6%)
Radial	42 (79.2%)
Access laterality	
Right	47 (88.7%)
Left	6 (11.3%)
Duration, minutes	102.4 ± 66.8
Contrast dose, mL	80.5 ± 31.6
Fluoroscopy time, minutes	43.6 ± 31.1
Reference air kerma, mGy	2114.0 ± 1349.4
Verapamil use	40 (75.5%)
Technical success with Vecta46	51 (96.2%)
Complications	
Intra-procedural	3 (5.7%)
Post-procedural	2 (3.8%)
Vecta46 catheterized region	0 (0.0%)

## Discussion

This is the first retrospective analysis of patients undergoing various neurovascular procedures using the Vecta46 IMC. Although previous reports, presented as abstracts, have documented successful placement of the Vecta46 catheter in the proximal MMA trunk for all cases of MMA embolization (21 cases) and safe navigation to the supraclinoid ICA segment for Surpass Evolve (Stryker Corporation) flow diversion treatment (nine cases), these are the only available data on the Vecta46 IMC [4-5]. Based on the results of our study with 51 successful cases (96.2%), we demonstrate that the Vecta46 IMC is a safe and effective tool for treating aneurysms, cSDHs, acute ischemic strokes, dAVFs, and AVMs. 

In the cases presented, the Vecta46 IMC aided in the insertion and navigation of appropriately sized interventional devices within the neurovascular system and served as a conduit for retrieval devices. Interestingly, Fuga et al. found that the use of IMC in coil embolization of unruptured cerebral aneurysms resulted in a significantly higher rate of Raymond-Roy Occlusion Classification class 1 after treatment [6]. While Vecta46 can be used as a guide for IMC, it can also be used for aspiration for mechanical thrombectomy. 

The Vecta46 IMC is available in three catheter lengths (125, 146, and 160 cm), a mini-triaxial access system compatible with 0.058” guides and 0.027” microcatheters, and supports angiographic injections through 0.070” guide catheters. Key features include an atraumatic tip for distal navigation, an 11 cm distal flex zone for improved maneuverability, and a 25 cm hydrophilic coating for smooth vessel passage. The full-length polytetrafluoroethylene (PTFE) liner ensures proper delivery, while the nitinol cross coil in the proximal segment provides support and pushability. These factors contribute to its navigability and compatibility with various microcatheters and guidewires and are crucial for effectively reaching distal occlusions and providing stable support during intricate procedures.

Aneurysm treatment comprised 30 cases (56.6%) where the Vecta46 IMC was used, making it the most common pathology. Among these, flow diversion was the most used treatment method (56.7%) (n = 17). These cases had a 100% technical success rate, with only one case (6.67%) involving a non-flow-limiting focal right M1 dissection that required angioplasty. Importantly, this complication was not due to the use of the Vecta46 IMC and was not apparent on subsequent imaging after correction. These results are comparable to the AXS Catalyst 5 IMC, which achieved a technical success rate of 91.7% with Surpass flow diversion embolization [7]. Additionally, the combined use of the AXS Catalyst 5 and a Surpass flow diverter resulted in a 95% complete occlusion rate upon follow-up (6.3±3.8 months) [7]. Both the AXS Catalyst 5 and Vecta46 IMCs had no procedure-related morbidity or mortality [7].

Fast recanalization is crucial in stroke patients for improved clinical outcomes [8]. The Vecta46 IMC was used for stroke thrombectomy in 11.3% (six cases) of its applications, achieving an 83.3% technical success rate (five cases). In all cases, Vecta46 was used as a guide rather than as an aspirator due to the size of the target vessel. Its predicate devices, AXS Vecta71 and Vecta74 IMC, were primarily used as aspirators due to their larger distal (0.082 in and 0.083 in, respectively) and proximal (0.085 in and 0.087 in, respectively) outer diameters. Vecta71/74 IMCs achieved successful recanalization rates of 90% in nine cases, with one mortality case within the follow-up period [9]. However, future studies are needed to investigate these findings due to the small sample sizes presented. 

**Figure 2:**
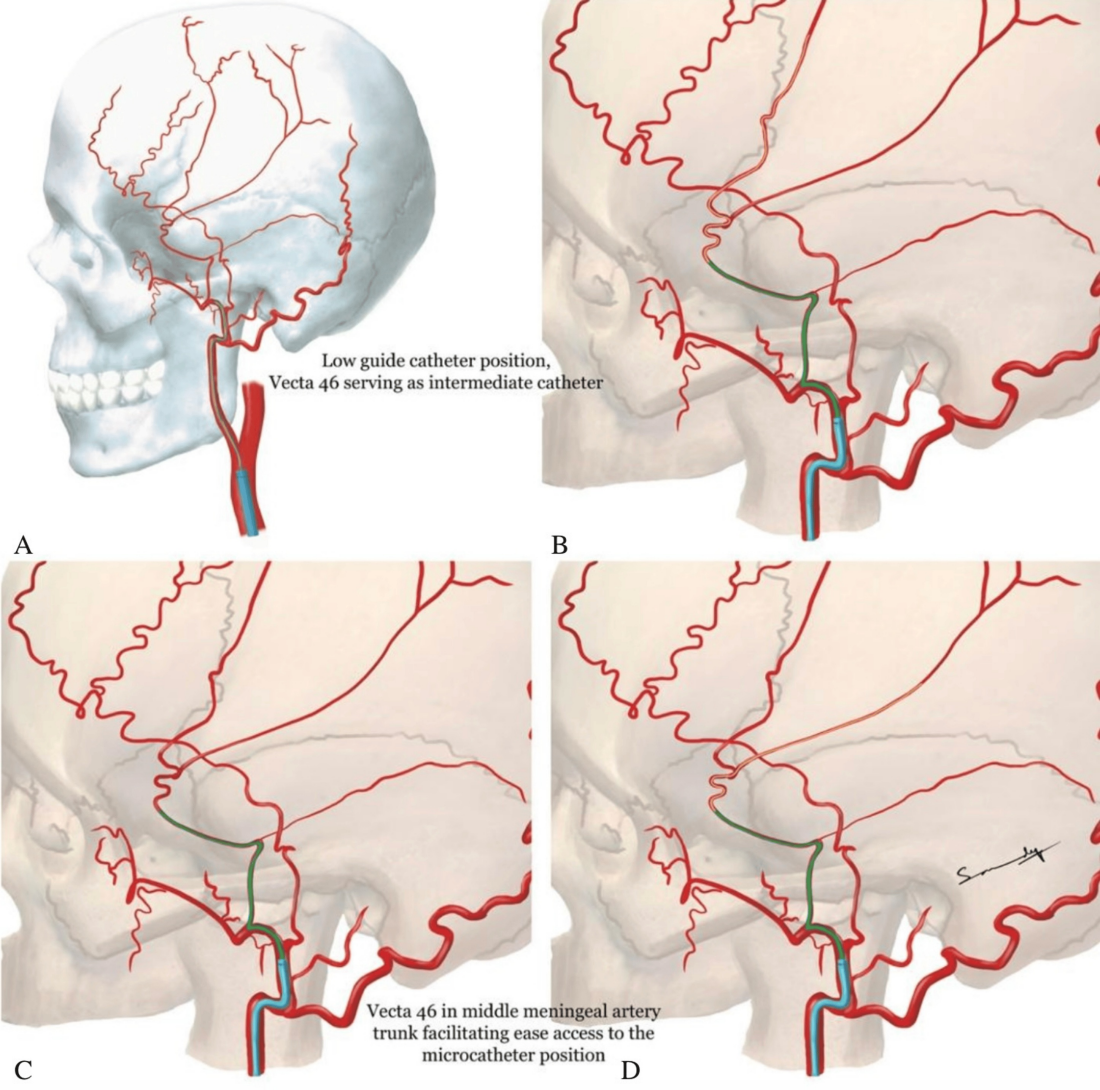
Anatomical visualization (A-D) The larger lumen of the Vecta46 intermediate catheter (green) facilitates excellent visualization of the entire middle meningeal artery anatomy and collaterals, allowing access to the microcatheter (orange) position. Image credits: Sandeep Kandregula, MD

An IMC’s navigability and support are helpful in safely reaching the MMA and delivering embolic agents, thus minimizing the risk of rebleeding and improving patient outcomes for MMA embolization in cSDH [10-11]. The Vecta46 IMC was 100% successful in reaching the MMA (10 cases). Vecta46 IMC is also comparable to the Zoom 45 catheter, a potential alternative assessed by Morsi et al., with a technical success rate of 93.8% (30/32) for MMA embolization cases and no complications [12]. The use of Vecta46 appears to be safe and effective for facilitating MMA embolization in patients with cSDHs and dAVFs. MMA embolization is quite feasible and, in many cases, has been performed without the use of intermediate catheters. However, the routine use of Vecta46 in our practice, as shown in this series, has a few benefits. Particularly with the transradial approach, left ECA catheterization is sometimes limited with the guide catheter. The Vecta46 provides distal purchase in the vessel and support of the microcatheter, overcoming the instability associated with lower positioning of the guide catheter (most often a .071” 6F guide catheter, Benchmark, or RIST). Secondly, optimal MMA embolization treatment has thus far been explored with individual catheterization of the frontal and parietal branches of the MMA [13]. Use of an IMC, particularly a low-profile one such as Vecta46, facilitates use of liquid embolic for a safer, direct microcatheter withdrawal; rapid catheterization of the second branch; and adjunct infusion such as the 5% dextrose in water (D5W) push technique [14-15]. Lastly, its larger lumen compared to standard microcatheters facilitates excellent visualization of the entire MMA anatomy and collaterals (Figure 2), which enhances the safety of subsequent embolization.

Neuroendovascular devices have revolutionized the treatment of cerebrovascular diseases, offering minimally invasive solutions that significantly improve patient outcomes. However, continuous post-market surveillance is essential to ensure the safety and efficacy of these devices as they are implemented in clinical practice. The data from post-market studies are invaluable, providing insights that guide the development and approval of future neuroendovascular technologies. Notably, the high rate of transradial access (79.2%) observed in this cohort underscores the growing preference for this approach in neurointerventional procedures, reflecting its benefits in terms of patient comfort and reduced complication rates [16]. These findings highlight the dynamic nature of neuroendovascular therapy and the importance of ongoing monitoring and adaptation in the field. 

The study's limitations include its retrospective nature and the relatively small sample size of 53 cases. Additionally, the lack of a control group prevents direct comparison with other IMCs. Furthermore, thrombectomy efficacy claims are limited to guide-catheter use; aspiration utility remains unstudied. Future prospective studies are needed to further validate these findings.

## Conclusions

The Vecta46 IMC demonstrated both effectiveness and safety across a variety of endovascular procedures in this real-world patient cohort. Continuous monitoring and assessment of its safety profile are crucial to ensure sustained clinical efficacy and to guide future innovations in neuroendovascular therapy. Preliminary data demonstrate the success of this intervention. Further studies in a prospective manner are potentially warranted to better understand in greater detail the outcomes of this catheter, as well as comparisons to other established IMCs. Additionally, multi-center studies should aim to evaluate long-term outcomes and cost efficiency.
